# Contrast-Enhanced Mammography in the Diagnosis of Breast Angiosarcoma

**DOI:** 10.1155/2021/5542786

**Published:** 2021-08-12

**Authors:** Maya Grisaru Kacen, Nikhil Sangle, Anat Kornecki

**Affiliations:** ^1^Department of Medical Imaging, Breast Division, St. Joseph's Health Care, Western University, London Ontario, Canada; ^2^Department of Pathology and Laboratory Medicine, London Health Sciences Center & St. Joseph's Health Care, London Ontario, Canada

## Abstract

A 60-year-old female presented for further assessment of a new right breast lump (November 2020). She had a history of a stage I (T1bN0M0) right breast invasive mammary carcinoma, grade 2 (score 7/9) with receptors ER/PR-negative, HER2/neu-positive, diagnosed four years prior to her current presentation. At that time, she was treated with a right breast lumpectomy and local radiation. Breast assessment with contrast-enhanced mammography showed new skin thickening with associated enhancement within the palpable region. Histology of subsequent ultrasound-guided biopsy found radiation-induced breast angiosarcoma. Breast angiosarcoma is a rare entity that represents less than 1% of all breast cancers. To our knowledge, this is the first case describing the imaging findings of breast angiosarcoma on contrast-enhanced mammography.

## 1. Introduction

Breast angiosarcoma is a rare entity associated with poor prognosis, accounting for 1 in 2000 cases of primary breast cancers [1]. The appearance on mammogram and ultrasound is nonspecific with high false-negative rates, and as a result, contrast-enhanced MRI is the imaging investigation of choice for evaluation of this rare condition [1–3].

Contrast-enhanced mammography (CEM) is an emerging breast imaging modality that combines two-dimensional (2D) digital mammography with iodine contrast administration. The exam utilizes a dual-energy technique, consisting of low-energy (LE) images, which are equivalent to 2D digital mammography, and high-energy (HE) images, which capture the information generated by the iodine and are nondiagnostic. The interpretation of CEM is based on the LE images and recombined images, which are generated by subtraction of LE images from the HE images. With a sensitivity that approaches that of MRI [4], CEM provides similar information more rapidly and at a lower cost [4, 5]. However, the reporting radiologist must be familiar with the presentation of different pathologies on all imaging modalities.

This case study offers the opportunity to learn about the appearance of radiation-induced breast angiosarcoma on CEM.

## 2. Patient Information

A 60-year-old female with a history of previously treated right breast carcinoma presented to our breast care centre with new complaints of right breast skin discoloration and a palpable lump within the region of the previous lumpectomy site.

Her previous breast cancer was stage I (T1bN0M0), grade 2 (score 7/9), negative for estrogen and progesterone hormone receptors, and positive for HER2/neu. Treatment consisted of a lumpectomy, adjuvant TC (cyclophosphamide) chemotherapy, and Herceptin. This was followed by local breast radiation therapy of 4250 cGy in 16 fractions completed by September 2017, three years prior to her new palpable concerns.

## 3. Clinical Findings

The patient's physical examination was conducted by a breast surgeon. The patient presented with right breast erythematous skin discoloration associated with multiple dermal lesions extending medially with the total region of involvement spanning over 8 cm. No palpable lymph nodes were noted on physical examination.

## 4. Timeline

In early November 2016, the patient presented with a palpable lump within the right breast. By the end of November 2016, she was diagnosed with right breast invasive mammary carcinoma, grade 2 (score 7/9), ER/PR-negative, and HER2-positive receptors. After her right breast lumpectomy in January 2017, she was treated with chemotherapy and HER2 inhibitor targeted therapy (Taxotere, cyclophosphamide, and Herceptin), followed by 16 fractions of radiation therapy which were completed in September 2017. She presented for her yearly routine mammographic surveillance in September 2017, August 2018, and August 2019. Her surveillance appointment for August 2020 was postponed due to COVID-19, and by November 2020, she presented with new symptoms and was diagnosed with angiosarcoma. Right breast modified mastectomy was completed in March 2021.

## 5. Diagnostic Assessment

**Figure 1:**
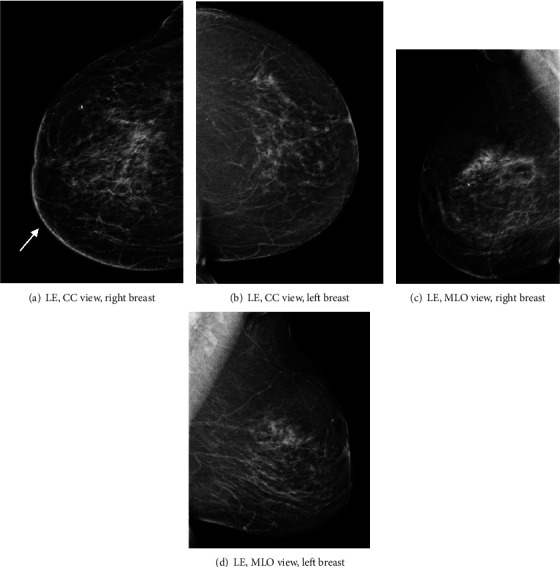
(a–d) Bilateral LE images in a craniocaudal (CC) and medial oblique (MLO) views. Posttreatment changes at the right inner breast are best appreciated on the CC view (arrow, (a)). A separate tissue marker is demonstrated in the right upper outer quadrant is related to a previous benign biopsy.

**Figure 2:**
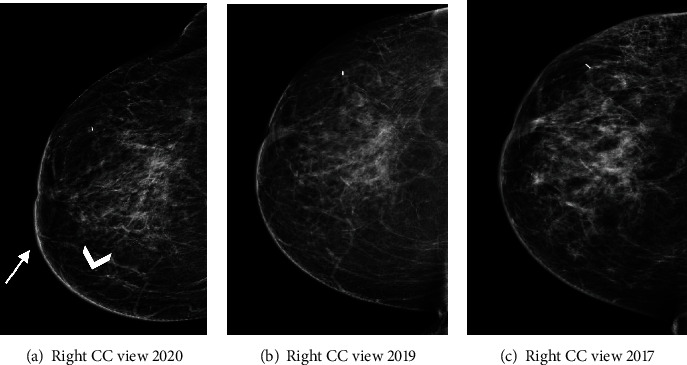
(a–c) Right LE image in a craniocaudal view (a) compared to the previous craniocaudal view from 2D mammography exams from one year ((b), 2019), and 3 years ((c), 2017) before show new mild skin thickening (arrow, (a)) and trabecular thickening (arrowhead, (a)).

**Figure 3:**
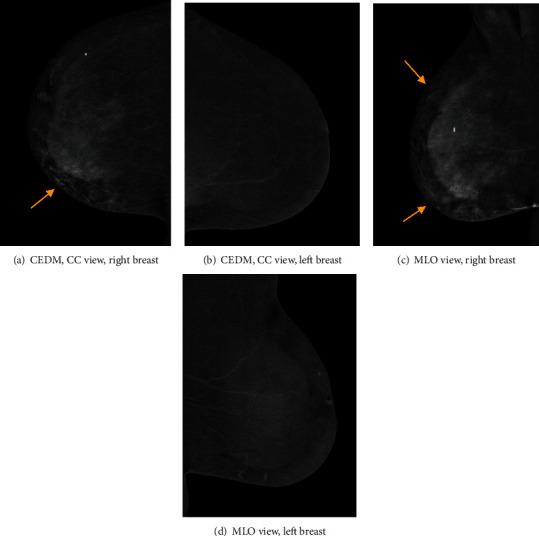
(a–d) Bilateral recombined images in a craniocaudal (CC) and medial oblique (MLO) views. There is minimal background parenchymal enhancement. Marked nonmass enhancement is noted predominantly within the inner aspect of the right breast, especially in the subcutaneous peripheral areas (yellow arrows, (a, c)).

**Figure 4:**
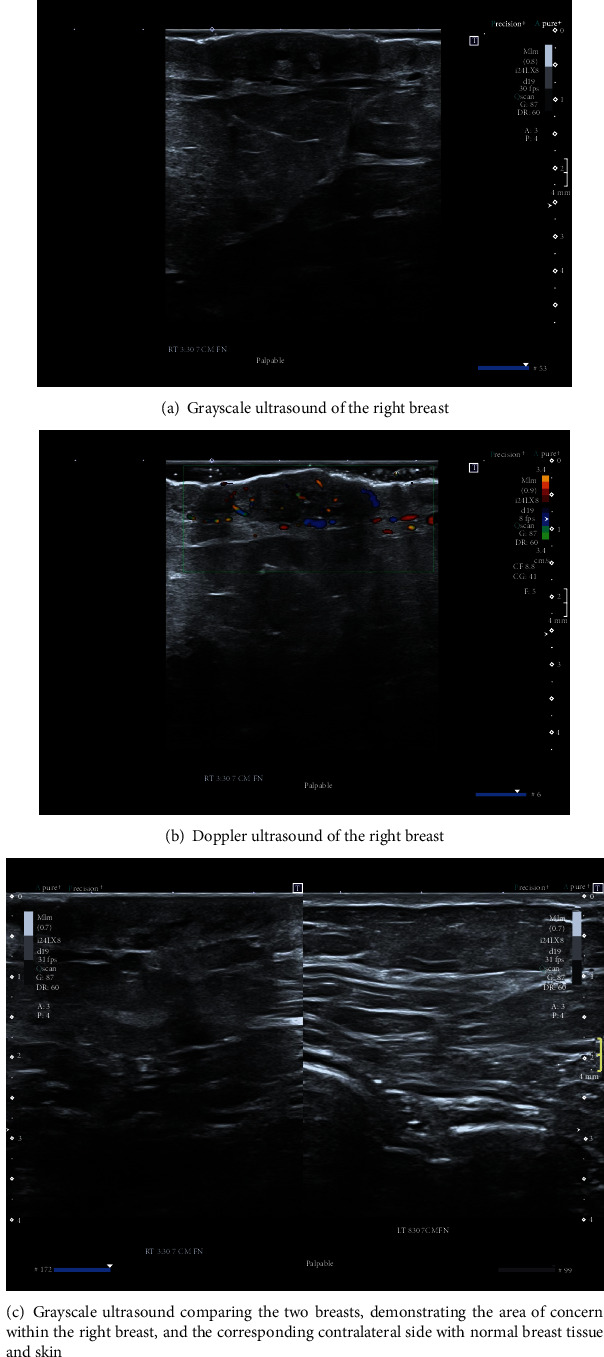
(a–c) Selected ultrasound exam on the right breast shows marked skin thickening measuring up to 7.2 mm, in the area of concern (a), associated with increased flow (b).

**Figure 5:**
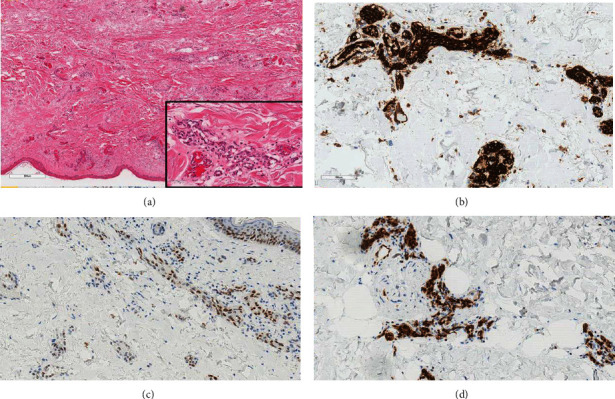
(a–d) Hematoxylin and eosin (H&E) stained section ((a), ×40 magnification) showing skin with underlying dermis containing an infiltrative vasoformative neoplasm. The endothelial cells lining these vascular spaces are cytologically atypical (inset, H&E ×400 magnification). Immunohistochemistry staining (×200 magnification) showed diffuse positivity in the malignant cells for CD31 (b), patchy staining for C-MYC (c), and nuclear immunoreactivity for ERG (d).

**Figure 6:**
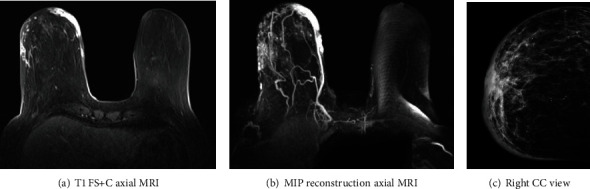
(a–c) This is a different case. An 80-year-old patient with remote history of right breast lumpectomy and radiation treatment due to breast malignancy. Patient presented with similar clinical symptoms of skin changes and discoloration. Axial T1 with fat suppression (FS), postcontrast injection (+C) MRI (Figure 6(a)), and MIP reconstruction (Figure 6(b)) demonstrating marked skin thickening and enhancement. Corresponding right CC mammographic view (Figure 6(c)) demonstrating marked skin thickening with no discreet mass within the breast.

Contrast-enhanced mammography was performed following the administration of intravenous 87 ml Omnipaque 350 mg. Bilateral LE images (Figures 1(a)–1(d)) showed posttreatment changes at the right upper inner quadrant. Comparison to previous exams from one and three years prior showed a new mild increase in skin thickening and trabecular thickening in the right inner breast (Figures 2(a)–2(c)). Recombined views (Figures 3(a)–3(d)) showed minimal bilateral background parenchymal enhancement (BPE) with marked nonmass enhancement noted on the right breast, subcutaneous region. Targeted the US of the right breast demonstrated marked skin thickening in the area of concern, associated with increased vascularity (Figures 4(a)–4(c)). This demonstrates marked skin thickening with no corresponding intraparenchymal breast lesion, similar to this case. Pathological slides images are also provided (Figures 5(a)–5(d)). MRI and mammography images from a different patient are provided for teaching purposes (Figures 6(a)–6(c)).

Ultrasound-guided biopsy was performed targeting the subcutaneous skin thickening, using a 14-gauge core biopsy needle. Pathology results confirmed an infiltrative, cytologically malignant, vasoformative neoplasm within the sampled tissues. The lesion cells stained positively for CD31, ERG, and c-Myc. The c-Myc immunopositivity is characteristic of radiation-associated angiosarcoma.

### 5.1. Therapeutic Intervention and Follow-Up

Since the patient had CEM, DCE-MRI was not necessary. Staging chest and abdominal CT study was obtained in January 2021 with no evidence of distant metastasis. PET-CT was not warranted. The patient received chemotherapy and was scheduled for a right modified radical mastectomy, using a skin flap for skin closure due to the large defect. The postsurgical pathology report described a 23.3 × 20 × 5.5 cm breast tissue specimen with irregular skin lesions composed of multiple confluent red papules extending over an area of 16 × 12.8 cm. Histologically, there was an infiltrative, cytologically malignant, vasoformative neoplasm present within the dermis and superficial subcutis. The nipple and areola were involved. Mitotic activity was not observed. The lesional cells stained diffusely positively for CD31 and ERG, while C-MYC showed patchy positivity. All histologically confirmed residual radiation-induced angiosarcoma showed minimal to no histological response to preoperative neoadjuvant chemotherapy (paclitaxel). The surgical skin resection margins were at least 1.0 cm from the angiosarcoma.

## 6. Discussion

Angiosarcoma of the breast is a rare entity which accounts for less than 1% of all breast malignancies and has a poor prognosis [2, 3, 6]. Primary and secondary forms of angiosarcoma are clinically distinct. Secondary angiosarcomas are usually induced either by radiation therapy or are detected in the setting of lymphedema. Angiosarcoma, in the setting of lymphedema, is more likely to occur in breast cancer patients who underwent radical mastectomy [1, 6, 7]. Radiation-induced angiosarcoma generally occurs after breast conservation surgery and radiation therapy and only rarely occurs after mastectomy. The average time between radiation therapy and the development of angiosarcoma is six years [1, 6]. However, several reports indicate that a diagnosis of angiosarcoma may occur as early as one to two years or as late as 41 years after treatment [6]. The mean age of presentation is in the late 60s [3, 6]. It usually affects the dermis of the breast within the radiation field but may occasionally develop within the breast parenchyma [6]. On clinical presentation, the breast can be firm on palpation and there may be associated skin discoloration ranging from blue, red, and purple [1, 3, 5, 6]. Dimpling of the skin is sometimes present as an indication of tissue congestion.

Mammography and ultrasound of radiation-induced angiosarcomas do not have characteristic pathognomonic imaging features. Imaging findings may be as indolent as mild skin thickening and trabecular changes, as seen in our case. Since these changes are expected in a patient with prior lumpectomy and radiation treatment, one must be able to detect even the mildest interval differences. Angiosarcomas characteristically result in high signal on T2 and low signal on T1 DCE-MRI-weighted images. Some studies report a rapid enhancement followed by rapid washout [8], while others report prolonged enhancement with no washout [2–6]. Given the overlapping reported kinetic curves, a malignant entity should still be considered in the differential diagnosis [6] keeping in mind that noncancerous, postirradiation breast tissue changes are not expected to enhance on either CEM or DCE-MRI three years after treatment.

In our department, contrast-enhanced mammography is offered routinely to provide rapid assessment in different clinical settings, including symptomatic patients. In the present case, CEM demonstrated minimal BPE, as expected for the patient's age group. As with DCE-MRI, decreased BPE on CEM improves specificity (9). While CEM is not a dynamic scan, the kinetic curve for angiosarcoma is not specific anyway, as discussed above. The main disadvantage of CEM, compared to DCE-MRI, is providing only 2D views which makes localizing and distinguishing subcutaneous enhancement from BPE more difficult. In our case, decreased BPE on the contralateral breast with marked asymmetric enhancement in the peripheral subcutaneous regions (Figures 3(a)–3(d)), along with new skin thickening, indicated to us that this was an abnormal exam. The ultrasound confirmed the feasibility of ultrasound-guided biopsy of a skin lesion with no evidence of intraparenchymal breast tissue mass. Angiosarcoma, inflammatory process (benign and malignant), and lymphoma were included in the differential diagnoses.

The prognosis of angiosarcoma depends on the tumour size, histological grade, and presence of metastasis at the time of presentation. Early diagnosis may play a key role in improving the morbidity and mortality of this condition. Therefore, secondary breast angiosarcomas should be considered in the differential diagnosis when it is clinically appropriate. The combination of new skin thickening seen on LE images associated with enhancment seen on recombined views and patient medical history, made us consider radiation-induced breast angiosarcoma in the differential diagnosis. The ability to perform second look ultrasound, ultrasound guided biopsy and CEM on the same day, made it possible for us to reach the final diagnosis more rapidly. This points to the main advantages of CEM in comparison to DCE-MRI.

Due to the infrequent incidence of breast angiosarcoma, there are no randomized trials comparing wide local excision and breast-conserving approach with mastectomy as treatment options. Given the aggressiveness and poor prognosis of this disease, total mastectomy with or without axillary node dissection is the preferred surgical treatment. The necessity for axillary node dissection is unclear since the hematogenous spread is more common in angiosarcomas than the lymphatic spread [8]. The reported median survival period ranges from 14.5 to 37 months, with a 5-year survival rate of 15% [6, 8].

## 7. Conclusion

This rare case of radiation-induced breast angiosarcoma in a symptomatic patient could have been missed by routine mammography alone, or the diagnosis could have been delayed if breast MRI was required. This is an example where a CEM study can replace DCE-MRI. The main imaging finding that raised concern for breast angiosarcoma in this case was the abnormal skin enhancement that was appreciated on the CEM which led to further assessment with targeted US and US-guided biopsy. This rare diagnostic case can be added to the multitude of studies showing the advantages of CEM in the diagnostic setting by allowing rapid diagnosis.
